# Including myositis-specific autoantibodies improves performance of the idiopathic inflammatory myopathies classification criteria

**DOI:** 10.1093/rheumatology/kez253

**Published:** 2019-06-25

**Authors:** Fergus To, Matthew J. S. Parker, Clara Ventín-Rodríguez, James B. Lilleker, Hector Chinoy

**Affiliations:** 1 Division of Rheumatology, University of British Columbia, Vancouver, British Columbia, Canada; 2 Department of Rheumatology, Institute of Rheumatology and Orthopaedics, Royal Prince Alfred Hospital, Camperdown, Sydney, NSW, Australia; 3 University of Sydney, Camperdown, Sydney, NSW, Australia; 4 Complejo Hospitalario Universitario de A Coruña, A Coruña, Spain; 5 Centre for Musculoskeletal Research, Division of Musculoskeletal and Dermatological Sciences, School of Biological Sciences, Faculty of Biology, Medicine and Health, Manchester Academic Health Science Centre, The University of Manchester, Manchester, UK; 6 Manchester Centre for Clinical Neurosciences, Salford Royal NHS Foundation Trust, Salford, UK; 7 National Institute for Health Research Manchester Biomedical Research Centre, Manchester University NHS Foundation Trust, The University of Manchester, Manchester, UK; 8 Department of Rheumatology, Salford Royal NHS Foundation Trust, Manchester Academic Health Science Centre, Salford, UK

**Rheumatology key message**

- Including additional myositis-specific autoantibodies into the EULAR/ACR classification criteria identifies idiopathic inflammatory myopathy patients and subtypes more accurately.

Sir, The 2017 EULAR/ACR classification criteria for idiopathic inflammatory myopathies (IIM) were developed to identify homogeneous populations of IIM patients for research purposes [1, 2]. The presence or absence of certain disease features contribute to an IIM probability score, with ⩾50% indicating ‘possible’, ⩾55% ‘probable’ and ⩾90% ‘definite’ IIM. Using the ‘probable IIM’ probability cut-off, sensitivity remains high (93%) [1]. However, while classification as ‘definite IIM’ is the suggested threshold for inclusion in studies where high specificity levels are required, sensitivity is lower (around 70%), limiting the number of patients eligible for enrolment [1].

Those with ‘definite IIM’ or ‘probable IIM’ can be further distinguished using a classification tree into one of four IIM subtypes: PM, IBM, amyopathic dermatomyositis and DM. As immune-mediated necrotizing myopathy (IMNM) was only recently recognized as a distinct entity, only small numbers of these cases were included in the classification design process. The authors were thus unable to distinguish PM from IMNM in the classification tree [1, 3].

As highlighted by Lundberg and Tjärnlund, another limitation of the criteria is the limited use of myositis-specific autoantibodies (MSAs), with only anti-Jo1 status included in the final criteria [4]. As the project to define these criteria commenced over a decade ago, many MSAs were either undiscovered or their detection assays were not widely accessible, preventing inclusion [1, 4]. However, recent years have seen a revolution in the availability of MSA testing, with highly specific and reliable line blot immunoassays commercially available and able to test for a broad complement of MSAs simultaneously.

It is considered that integration of a wider repertoire of MSAs into updated classification criteria might improve performance both in terms of case definition and in assigning IIM subtype. To evaluate this, we conducted a study of patients in our IIM cohort where a panel of MSA results, in addition to anti-Jo1, were available. We identified all adult patients (⩾18 years at disease onset) with a physician-verified diagnosis of IIM. Details of data source and case ascertainment are available in the supplementary material, section Case ascertainment, available at *Rheumatology* online. The EULAR/ACR criteria were applied to each case and results were categorized using the suggested cut-points into non-IIM, possible IIM, probable IIM and definite IIM. We then identified all patients with a non-anti-Jo1 MSA including anti-PL7, anti-PL12, anti-EJ, anti-OJ, anti-Mi2, anti-MDA5, anti-SAE1, anti-transcription intermediary factor 1γ, anti-NXP2 and anti-signal recognition particle using a line blot immunoassay (EUROLINE Inflammatory Myopathies 16 Ag, Euroimmun, Lubeck, Germany). This assay has not been fully validated yet, but has high reported specificity for IIM [5]. Anti-3-hydroxy-3-methyl-glutaryl-coenzyme A reductase was also identified via ELISA. The same criteria including classification tree were then reapplied, with the non-anti-Jo1 MSAs assigned the same weight as an anti-Jo1 antibody.

We identified 309 patients with an average age of 55.6 years, of whom 62.8% were female. Of these, 27/309 (8.7%) possessed anti-Jo1 antibodies, while 78/309 (25.2%) were negative for anti-Jo1 antibodies, but had an alternative MSA. This work forms part of a national quality improvement project aimed at accurate identification of IIM cases for development of specialized disease commissioning and service planning. Given this context, approval for the conduct of the project was granted without a recommendation to seek more formal ethics authorization. In the non-anti-Jo1 MSA-positive subgroup, according to the EULAR/ACR criteria, 57/78 (73.1%) had ‘definite IIM’, 13/78 (16.7%) ‘probable IIM’, 0/78 ‘possible IIM’ and 8/78 (10.3%) ‘non-IIM’. When other MSAs were given the same weight as an anti-Jo1 in the antibody criterion, classification of definite IIM increased to 75/78 (96.2%) patients. Those with probable IIM reduced to 3/78 (3.8%) and no patients were defined as ‘non-IIM’ (Table 1).

**Table 1:** The relationship between EULAR/ACR classification criteria with and without inclusion of non-anti-Jo1 MSAs

	Classification with non-anti-Jo1 MSAs included				
Current classification	Definite IIM	Probable IIM	Possible IIM	Non-IIM	Totals
Definite IIM	57	0	0	0	57 (73.1%)
Probable IIM	13	0	0	0	13 (16.7%)
Possible IIM	0	0	0	0	0
Non-IIM	5	3	0	0	8 (10.3%)
Totals	75 (96.2%)	3 (3.8%)	0	0	

IIM: idiopathic inflammatory myopathy; MSA: myositis-specific autoantibody.

Currently, MSAs are not included in the classification tree used to define IIM subtypes. In our cohort, 25/78 (32.1%) patients were subtyped as PM and had an MSA other than anti-Jo1. Fifteen of 78 (19.2%) patients had a diagnosis of IMNM with either anti-3-hydroxy-3-methyl-glutaryl-coenzyme A reductase or anti-signal recognition particle antibodies and met 2017 European Neuromuscular Centre criteria for IMNM [3]. A further 8/78 (10.3%) cases had antisynthetase syndrome with a relevant antisynthetase antibody. One of 78 (1.3%) patients had dermatomyositis *sine dermatitis* with an anti-NXP2 antibody.

We highlight improved performance of the EULAR/ACR classification criteria after inclusion of widely available MSA results, building on the experience of others who have examined the effect of including antisynthetase antibodies [6]. We have demonstrated that including non-anti-Jo1 MSAs increases the likelihood of classifying patients as ‘definite IIM’ or ‘probable IIM’, facilitating accurate diagnosis and inclusion of patients into clinical trials and research studies. Additionally, inclusion of MSAs into the classification tree can more precisely define IIM subtypes, reducing the number of patients incorrectly classified as PM and potentially supporting the use of targeted treatments for individual subgroups and facilitating accurate inclusion in subgroup-specific clinical trials. We suggest that an additional layer of the classification tree referring to MSA status is added at the point where patients are currently subtyped as PM. Elements from the 2017 European Neuromuscular Centre criteria for IMNM may be integrated into this. Limitations to the study include its retrospective single-centre design and the absence of a comparator group. Finally, the specificities of all MSAs are not yet fully determined [7].

*Funding*: This work was supported by a grant from the Medical Research Council (MR/N003322/1). This report includes independent research supported by the National Institute for Health Research (NIHR) Biomedical Research Centre Funding Scheme. The views expressed in this publication are those of the authors and not necessarily those of the National Health Service, the NIHR or the Department of Health.

*Disclosure statement*: The authors have declared no conflicts of interest.

## Supplementary Material

kez253_Supplementary_Data
