# Supplementary material for: Including myositis-specific autoantibodies improves performance of the idiopathic inflammatory myopathies classification criteria
Source: Rheumatology (Oxford). 2019 Jun 25;58(12):2331–3. doi: 10.1093/rheumatology/kez253 (PMC6880850; doi:10.1093/rheumatology/kez253)
Supplement: kez253_Supplementary_Data [file kez253_supplementary_data.docx]

**SUPPLEMENTARY MATERIAL**

**Case ascertainment**

All patients are from Salford Royal NHS Foundation Trust (SRFT)’s neuromuscular service. Three stages of case ascertainment were employed. First, an International Classification of Diseases, 10^th^ revision search for IIM was conducted for all SRFT inpatients between January 2007-December 2016. Secondly, all new patients referred to the SRFT outpatient clinic was manually identified during that time. Finally, all patients with MSAs assays requested between January 2007-July 2018 were identified and their medical records were reviewed. All patients diagnosed with IIM by their treating physicians were identified. These data were merged and duplicates were removed.
